# Excavatoids E and F: Discovery of Two New Briaranes from the Cultured Octocoral *Briareum excavatum*

**DOI:** 10.3390/md7030472

**Published:** 2009-09-23

**Authors:** Ping-Jyun Sung, Bo-Yuan Chen, Mei-Ru Lin, Tsong-Long Hwang, Wei-Hsien Wang, Jyh-Horng Sheu, Yang-Chang Wu

**Affiliations:** 1 Graduate Institute of Marine Biotechnology and Department of Life Science and Graduate Institute of Biotechnology, National Dong Hwa University, Checheng, Pingtung 944, Taiwan; E-Mail:chenpaul0205@hotmail.com (B.-Y.C.); 2 National Museum of Marine Biology & Aquarium, Checheng, Pingtung 944, Taiwan; E-Mails:linmeiru@hotmail.com (M.-R.L.);whw@nmmba.gov.tw (W.-H.W.); 3 Department of Marine Biotechnology and Resources and Asia-Pacific Ocean Research Center, National Sun Yat-sen University, Kaohsiung 804, Taiwan; E-Mail:sheu@mail.nsysu.edu.tw (J.-H.S.); 4 Graduate Institute of Natural Products, Chang Gung University, Taoyuan 333, Taiwan; E-Mail:htl@mail.cgu.edu.tw (T.-L.H.); 5 Graduate Institute of Natural Products, Kaohsiung Medical University, Kaohsiung 807, Taiwan

**Keywords:** excavatoid, briarane, octocoral, Briareum excavatum, human neutrophil

## Abstract

Two new briarane-related diterpenoids, designated as excavatoids E (**1**) and F (**2**), were isolated from the cultured octocoral *Briareum excavatum*. The structures of compounds **1** and **2** were established on the basis of extensive spectral data analysis. Briaranes **1** and **2** were found to exhibit moderate inhibitory effects on elastase release by human neutrophils.

## 1. Introduction

**Figure 1:**
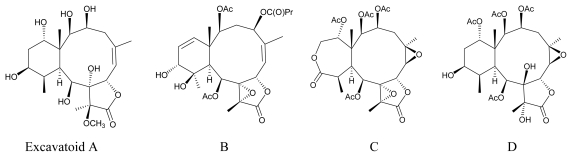
The Structures of Excavatoids A-D.

**Figure 2:**
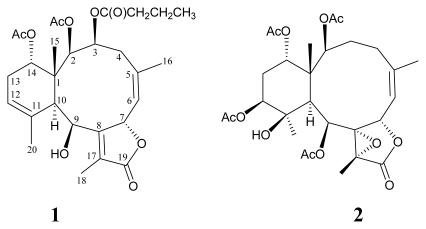
The Structures of Excavatoids E (**1**) and F (**2**).

In our continuing research on chemical constituents of marine invertebrates collected in Taiwan waters, a series of interesting and complex briarane-type diterpenoid derivatives (3,8-cyclized cembranoids), have been isolated from the octocorals belonging to the genus *Briareum* [1–21], *Ellisella* [18,22–25], and *Junceella* [16,26–38], and the compounds of this type were proven to possess various interesting bioactivities [39–41]. Because of its interesting and potential chemical constituents and a series of new briarane metabolites, including briaexcavatins I-Z [16–20] and excavatoids A-D [21] (Figure 1), the octocoral *B. excavatum* was transplanted to the National Museum of Marine Biology & Aquarium (NMMBA), Taiwan. We report herein the isolation, structure determination, and bioactivity of two new briaranes, excavatoids E (**1**) and F (**2**) (Figure 2), resulting from further studies on the chemical constituents of cultured *B. excavatum*. The structures of compounds **1** and **2** were established by extensive spectroscopic methods and these two metabolites have displayed moderate inhibitory effects on elastase release by human neutrophils.

## 2. Results and Discussion

Excavatoid E (**1**) was obtained as a white powder and the molecular formula of **1** was determined to be C_28_H_38_O_9_ by analysis of 13C- and 1H-NMR data, in conjunction with DEPT results (Table 1); this conclusion was further confirmed by HRESIMS with *m/z* 541.2415 (calcd. for C_28_H_38_O_9_Na, 541.2413). This showed that **1** contained 10 degrees of unsaturation. Comparison of the ^1^H and DEPT data with the molecular formula indicated that there must be an exchangeable proton, requiring the presence of a hydroxy group, and this deduction was supported by a broad band in the IR spectrum at 3,463 cm^−1^. The IR absorptions of **1** also showed the presence of α,β-unsaturated γ-lactone (1,743 cm^−1^) and ester (1,740 cm^−1^) groups. From the ^13^C-NMR spectrum (Table 1), compound **1** was found to possess an *n*-butyryloxy group (*δ*_C_ 13.6, q; 18.0, t; 35.9, t; 172.3, s); two acetoxy groups (*δ*_C_ 21.2, q; 20.9, q; 170.4, s; 169.1, s), a γ-lactone moiety (*δ*_C_ 173.9, s, C-19), a tetrasubstituted olefin (*δ*_C_ 160.8, s, C-8; 127.6, s, C-17), and two disubstituted olefins (*δ*_C_ 141.9, s, C-5; 123.9, d, CH-6; 130.5, s, C-11; 123.4, d, CH-12). Thus, from the above data, seven degrees of unsaturation were accounted for, and compound **1** must be tricyclic.

Moreover, a methyl singlet (*δ*_H_ 1.36, 3H, s, H_3_-15), three vinyl methyls (*δ*_H_ 2.01, 3H, s, H_3_-18; 1.85, 3H, s, H_3_-16; 1.64, 3H, s, H_3_-20), two pairs of methylene protons (*δ*_H_ 3.46, 1H, dd, *J* = 15.6, 6.0 Hz; 2.04, 1H, d, *J* = 15.6 Hz, H_2_-4; 2.57, 1H, br d, *J* = 18.4 Hz; 2.15, 1H, m, H_2_-13), an aliphatic methine proton (*δ*_H_ 3.25, 1H, br s, H-10), five oxymethine protons (*δ*_H_ 6.07, 1H, d, *J* = 8.8 Hz, H-7; 5.45, 1H, d, *J* = 2.4 Hz, H-2; 5.08, 1H, dd, *J* = 6.0, 6.0 Hz, H-14; 4.98, 1H, br s, H-9; 4.98, 1H, dd, *J* = 6.0, 2.4 Hz, H-3), two olefin protons (*δ*_H_ 5.58, 1H, br s, H-12; 5.21, 1H, d, *J* = 8.8 Hz, H-6), and two acetyl methyls (*δ*_H_ 2.12, 3H, s; 1.99, 3H, s) were observed in the ^1^H-NMR spectrum of **1**. An *n-*butyryl group (*δ*_H_ 0.94, 3H, t, *J* = 7.6 Hz; 1.61, 2H, m; 2.22, 2H, t, *J* = 7.6 Hz) was also observed in the ^1^H-NMR spectrum and further confirmed by the ^1^H-^1^H COSY correlations and coupling constant analysis.

The gross structure of **1** was elucidated with the assistance of 2D NMR studies. From the ^1^H-^1^H COSY experiment of **1** (Table 2), it was possible to establish the separate spin systems that map out the proton sequences from H-2/3/4, H-6/7, and H-9/10. These data, together with the HMBC correlations between H-2/C-1, −3, −4; H-3/C-1; H_2_-4/C-3, −5, −6; H-6/C-4; H-7/C-5, −6, −8; H-9/C-1, −8; and H-10/C-9, established the connectivity from C-1 to C-10 in the 10-membered ring (Table 2). The vinyl methyl groups attached at C-5 and C-11 were confirmed by the HMBC correlations between H_3_-16/C-4, −5, −6; and H_3_-20/C-10, −11, −12, and further supported by the allylic couplings between H-6/H_3_-16 and H-12/H_3_-20, respectively. The vinyl methyl group attached at C-17 was also established by the HMBC correlations between H_3_-18 and C-8, −17, −19. The methylcyclohexene ring, which is fused to the 10-membered ring at C-1 and C-10, was elucidated by the ^1^H-^1^H COSY correlations between H-12/H_2_-13/H-14 and by the HMBC correlations between H-2/C-14; H-10/C-11; H-14/C-1, −2; and H_3_-20/C-10. The ring junction C-15 methyl group was positioned at C-1 from the HMBC correlations between H-2/C-15; H-14/C-15; and H_3_-15/C-1, −2, −10, −14. In addition, the HMBC correlations also revealed that two acetates should attach at C-2 and C-14, respectively. The remaining *n*-butyryloxy and hydroxy groups were positioned at C-3 and C-9 as indicated by analysis of ^1^H-^1^H COSY correlations and characteristic NMR signals analysis, although no HMBC correlation was observed between H-3 and the *n*-butyrate carbonyl.

**Figure 3:**
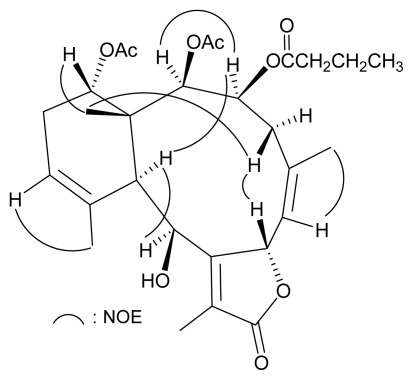
Selected NOE Correlations of **1**.

Based on previous studies, all naturally occurring briarane-type diterpenoids have the C-15 methyl group *trans* to H-10, and these two groups are assigned as β- and α-oriented, respectively, as shown in most briarane derivatives [42–44]. The relative stereochemistry of **1** was established from a NOESY experiment (Figure 3), in which the NOE correlations of H-10 with H-3 and H-9; and H-3 with H-2, indicated that these protons are situated on the same face and were assigned as *α* protons since the C-15 methyl is the β-substituent at C-1. H-14 was found to exhibit a correlation with H_3_-15 but not with H-10, revealing the β-orientation of this proton. One of the C-4 methylene protons (*δ*_H_ 3.46) exhibited a correlation with H_3_-15 and was assigned as H-4*β*, while the other was denoted as H-4*α* (*δ*_H_ 2.04). A correlation observed between H-4*β* and H-7, reflected the *β*-orientation of H-7. The NOESY spectrum showed correlations of H-6/H_3_-16 and H-12/H_3_-20, revealing the *Z* geometry of C-5/6 and C-11/12 double bonds in **1**.

The molecular formula of excavatoid F (**2**) was determined as C_28_H_38_O_12_ by its HRESIMS (*m/z* 589.2257, calcd. for C_28_H_38_O_12_Na, 589.2261). The IR spectrum showed bands at 3,487, 1,779, and 1,737 cm^−1^, consistent with the presence in **2** of hydroxy, γ-lactone, and ester groups. From the ^13^C-NMR data of **2** (Table 1), a trisubstituted olefin was deduced from the signals of two carbons at *δ*_C_ 146.2 (s, C-5) and 117.6 (d, CH-6). A methyl-containing tetrasubstituted epoxy group was confirmed from the signals of two quaternary oxygenated carbons at *δ*_C_ 70.6 (s, C-8) and 64.5 (s, C-17), and from the chemical shifts of a tertiary methyl group (*δ*_H_ 1.73, 3H, s, H_3_-18; *δ*_C_ 10.3, q, CH_3_-18) (Table 1). Moreover, five carbonyl resonances appeared at *δ*_C_ 170.9 (s, C-19), 170.6, 170.5, 169.5, and 168.1 (4 × s, ester carbonyls), confirming the presence of a γ-lactone and four esters in **2**. All the esters were identified as acetates by the presence of methyl resonances in the ^1^H-NMR spectrum at *δ*_H_ 2.19, 2.07, 2.05, and 2.00 (each 3H × s).

**Figure 4:**
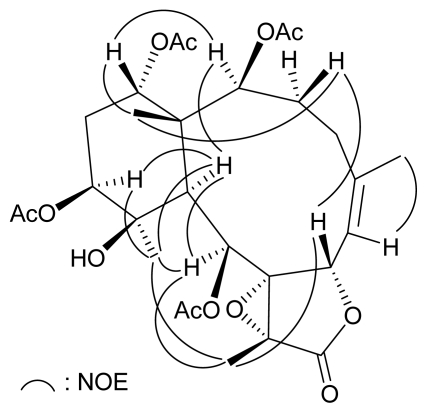
Selected NOE Correlations of **2**.

The planar structure of **2** was determined mainly by 2D NMR studies. The coupling information in the ^1^H-^1^H COSY spectrum of **2** enabled identification of the proton sequences H-2/3/4, H-4/6 (by allylic coupling), H-6/7, H-6/H_3_-16 (by allylic coupling), H-9/10, and H-12/13/14 (Table 2). These data, together with the correlations observed in an HMBC experiment of **2** (Table 2), the molecular framework of **2** could be further established. The HMBC correlations also indicated that the acetoxy groups should attach at C-2, −9, −12, and C-14. Thus, the remaining hydroxy group has to be positioned at C-11, as indicated by characteristic NMR signal analysis. The relative stereochemistry of **2** was elucidated from the NOE interactions observed in a NOESY experiment (Figure 4). In the NOESY spectrum of **2**, correlations were observed between H-10 with H-2, −9, −12, and H_3_-20, indicating that these protons should be positioned on the α face in **2** and Me-20 was positioned on the equatorial direction in the methylcyclohexane ring of **2**. One proton attaching at C-3 (*δ*_H_ 2.65) was found to exhibit a correlation with H_3_-15 and was assigned as H-3*β* proton. H-7 showed a correlation with H-3*β*, confirming the *β*-orientation for this proton. Furthermore, H_3_-18 was found to show correlations with H-9, H_3_-20, and H-7, and from molecular models, was found to be reasonably closed to H-9, H_3_-20, and H-7; therefore, H_3_-18 should be placed on the β face in the γ-lactone ring of **2**. The *Z*-configuration of the C-5/C-6 double bond was elucidated by an interaction between H-6 (*δ*_H_ 5.25) and H_3_-16 (*δ*_H_ 2.02). On the basis of the above results, the structure of **2**, including the relative configuration, was elucidated.

In previous study, several diterpenoid derivatives of potential medical interest were isolated from a cultured gorgonian coral *Erythropodium caribaeorum* [45]. Because all corals are claimed to be threatened species, we wanted to maintain and culture these interesting specimens as sources of new and interesting natural products in our continuing search for novel substances from marine organisms originally collected in Taiwan waters, in the hope of identifying extracts that exhibit bioactivity. Briaranes **1** and **2** displayed moderate inhibitory effects on elastase release by neutrophils, and briarane **1** exhibited weak inhibitory effects on superoxide anion generation by human neutrophils at 10 μg/mL, respectively (Table 3). Furthermore, these two compounds were not cytotoxic toward the CCRF-CEM (human T-cell acute lymphoblastic leukemia) and DLD-1 (human colon adenocarcinoma) cells (ED_50_ > 40 μg/mL). The possible bioactivity for these two compounds will be further studied if we can obtain enough material from the cultured *B. excavatum* in the future.

## 3. Experimental Section

### 3.1. General Experimental Procedures

Melting points were determined on a Fargo apparatus and were uncorrected. Optical rotation values were measured with a JASCO P-1010 digital polarimeter at 25 °C. Infrared spectra were obtained on a Varian Digilab FTS 1000 FT-IR spectrometer. The NMR spectra were recorded on a Varian Mercury Plus 400 FT-NMR at 400 MHz for ^1^H and 100 MHz for ^13^C, in CDCl_3_, respectively. Proton chemical shifts were referenced to the residual CHCl_3_ signal (*δ* 7.26 ppm). ^13^C-NMR spectra were referenced to the center peak of CDCl_3_ at *δ* 77.1 ppm. ESIMS and HRESIMS data were recorded on a Bruker APEX II mass spectrometer. Column chromatography was performed on silica gel (230–400 mesh, Merck, Darmstadt, Germany). TLC was carried out on precoated Kieselgel 60 F_254_ (0.25 mm, Merck) and spots were visualized by spraying with 10% H_2_SO_4_ solution followed by heating. HPLC was performed using a system comprised of a Hitachi L-7100 pump, a Hitachi photodiode array detector L-7455, and a Rheodyne 7725 injection port. A semi-preparative reverse phase column (Hibar 250 × 10 mm, LiChrospher 100 RP-18e, 5 μm, Merck) was used for HPLC.

### 3.2. Animal Material

Specimens of the octocoral *Briareum excavatum* were collected and transplanted to a 0.6-ton cultivating tank located in the NMMBA, Taiwan, in December 2003, and the material for this research work was collected from the tank in December 2006. This organism was identified by comparison with previous descriptions [46–48]. A voucher specimen was deposited in the National Museum of Marine Biology & Aquarium, Taiwan (NMMBA-CSC-001).

### 3.3. Extraction and Isolation

The freeze-dried and minced material of *B. excavatum* (wet weight 672 g, dry weight 270 g) was extracted with a mixture of MeOH and CH_2_Cl_2_ (1:1) at room temperature. The residue was partitioned between EtOAc and H_2_O. The EtOAc layer was separated on Sephadex LH-20 and eluted using MeOH/CH_2_Cl_2_ (2:1) to yield three fractions A-C. Fraction C was separated on silica gel and eluted using hexane/EtOAc (stepwise, 20:1-pure EtOAc) to yield fractions 1–9. Fraction C7 was separated by reverse phase HPLC, using the mixtures of MeOH, CH_3_CN, and H_2_O to afford briaranes **1** (2.9 mg, 65/1/34) and **2** (1.2 mg, 62/1/37).

Excavatoid E (**1**): white powder; mp 192–193 °C; [*α*]_D_^25^ + 2 (*c* 0.15, CHCl_3_); IR (neat) *ν*_max_ 3,463, 1,743, 1,737 cm^−1; 13^C-NMR (CDCl_3_, 100 MHz) and 1H-NMR (CDCl_3_, 400 MHz) data, see Table 1; ESIMS *m/z* 541 (M + Na)^+^; HRESIMS *m/z* 541.2415 (calcd. for C_28_H_38_O_9_Na, 541.2413).

Excavatoid F (**2**): white powder; mp 164–165 °C; [*α*]_D_^25^ −16 (*c* 0.06, CHCl_3_); IR (neat) *ν*_max_ 3,487, 1,779, 1,737 cm^−1; 13^C-NMR (CDCl_3_, 100 MHz) and 1H-NMR (CDCl_3_, 400 MHz) data, see Table 1; ESIMS *m/z* 589 (M + Na)^+^; HRESIMS *m/z* 589.2257 (calcd. for C_28_H_38_O_12_Na, 589.2261).

### 3.4. Human Neutrophil Superoxide Anion Generation and Elastase Release

Human neutrophils were obtained by means of dextran sedimentation and Ficoll centrifugation. Superoxide generation was carried out according to the procedures described previously [49,50]. Briefly, superoxide anion production was assayed by monitoring the superoxide dismutase-inhibitable reduction of ferricytochrome *c*. Elastase release experiments were performed using MeO-Suc-Ala-Ala-Pro-Valp-nitroanilide as the elastase substrate.

## Figures and Tables

**Table 1:** ^1^H and ^13^C-NMR data for Diterpenoids **1** and **2**.

	1		2	
Position	^1^H^a^	^13^C^b^	^1^H^a^	^13^C^b^
1		41.7 (s)^d^		46.3 (s)
2	5.45 d (2.4)^c^	74.9 (d)	5.03 d (7.2)	75.3 (d)
3*α*	4.98 dd (6.0, 2.4)	72.2 (d)	1.69 m	32.5 (t)
*β*			2.65 ddd (15.6, 15.6, 6.0)	
4*α*	2.04 d (15.6)	34.4 (t)	1.97 m	28.7 (t)
*β*	3.46 dd (15.6, 6.0)		2.50 m	
5		141.9 (s)		146.2 (s)
6	5.21 d (8.8)	123.9 (d)	5.25 d (9.2)	117.6 (d)
7	6.07 d (8.8)	79.2 (d)	5.32 d (9.2)	74.9 (d)
8		160.8 (s)		70.6 (s)
9	4.98 br s	68.4 (d)	5.86 d (2.0)	67.8 (d)
10	3.25 br s	45.2 (d)	2.20 d (2.0)	47.8 (d)
11		130.5 (s)		75.7 (s)
12	5.58 br s	123.4 (d)	4.90 dd (11.6, 5.2)	73.1 (d)
13*α*	2.15 m	28.0 (t)	1.89–1.97 m (2H)	25.6 (t)
*β*	2.57 br d (18.4)			
14	5.08 dd (6.0, 6.0)	75.6 (d)	4.87 dd (4.8, 2.0)	75.6 (d)
15	1.36 s	16.7 (q)	1.30 s	15.6 (q)
16	1.85 s	23.0 (q)	2.02 s	27.0 (q)
17		127.6 (s)		64.5 (s)
18	2.01 s	10.2 (q)	1.73 s	10.3 (q)
19		173.9 (s)		170.9 (s)
20	1.64 s	22.1 (q)	1.24 s	27.9 (q)
2-OAc		169.1 (s)		170.5 (s)
	2.12 s	20.9 (q)	2.00 s	21.3 (q)
9-OAc				168.1 (s)
			2.19 s	21.5 (q)
12-OAc				169.5 (s)
			2.07 s	21.1 (q)
14-OAc		170.4 (s)		170.6 (s)
	1.99 s	21.2 (q)	2.05 s	21.3 (q)
3-OCOPr		172.3 (s)		
	2.22 t (7.6)	35.9 (t)		
	1.61 m	18.0 (t)		
	0.94 t (7.6)	13.6 (q)		

a Spectra were recorded at 400 MHz at 25 °C.

b Spectra were recorded at 100 MHz at 25 °C.

c *J* values (in Hz) in parentheses.

d Multiplicity deduced by DEPT and HMQC spectra and indicated by usual symbols.

**Table 2:** The ^1^H-^1^H COSY and HMBC (H→C) Correlations for Diterpenoids **1** and **2**.

Position	1		2	
	^1^H-^1^H COSY	HMBC	^1^H-^1^H COSY	HMBC
H-2	H-3	C-1, −3, −4, −14, −15, acetate carbonyl	H_2_-3	C-1, −3, −4, −10, −14, −15, acetate carbonyl
H-3	H-2, H_2_-4	C-1	H-2, H_2_-4	C-1, −4
H-4	H-3	C-3, −5, −6, −16	H_2_-3, H-6	C-5, −6
H-6	H-7, H_3_-16	C-4, −16	H-4, H-7, H_3_-16	C-4
H-7	H-6	C-5, −6, −8	H-6	C-5, −6, −19
H-9	H-10	C-1, −8	H-10	C-1, −7, −8, −10, −17, acetate carbonyl
H-10	H-9	C-9, −11	H-9	C-1, −2, −8, −9, −11, −14, −15
H-12	H_2_-13, H_3_-20	n.o.^a^	H_2_-13	acetate carbonyl
H-13	H-12, H-14	n.o.	H-12, H-14	C-1, −11, −14
H-14	H_2_-13	C-1, −2, −13, −15, acetate carbonyl	H_2_-13	C-10, acetate carbonyl
H-15		C-1, −2, −10, −14		C-1, −2, −14
H-16	H-6	C-4, −5, −6	H-6	C-4, −5, −6
H-18		C-8, −17, −19		C-8, −17, −19
H-20	H-12	C-10, −11, −12		C-10, −11, −12

a n.o. = not observed.

**Table 3:** Inhibitory Effects of Compounds **1** and **2** on Elastase Release and Superoxide Anion Generation by Human Neutrophils in Response to fMet-Leu-Phe/Cytochalastin B.

	Elastase	Superoxide Anion
	
Compound	Inh.%	Inh.%
**1**	26.22 ± 0.50 ***	12.95 ± 6.99
**2**	30.63 ± 4.68 *	2.57 ± 1.11

Percentage of inhibition (Inh.%) at 10 *μ*g/mL concentration. Results are presented as mean ± S.E.M. (*n* = 2–3).

* *P* < 0.05,

** *P* < 0.01, and

*** *P* < 0.001 compared with the control value.
